# Thrombin Production and Human Neutrophil Elastase Sequestration by Modified Cellulosic Dressings and Their Electrokinetic Analysis

**DOI:** 10.3390/jfb2040391

**Published:** 2011-12-15

**Authors:** Judson Vincent Edwards, Nicolette Prevost

**Affiliations:** Southern Regional Research Center, United States Department of Agriculture, Agricultural Research Service, 1100 Robert E. Blvd, New Orleans, LA 70124, USA; E-Mail: nicolette.prevost@ars.usda.gov

**Keywords:** thrombin, human neutrophil elastase, cellulosic wound dressings, electrokinetic, hemostasis, inflammation

## Abstract

Wound healing is a complex series of biochemical and cellular events. Optimally, functional material design addresses the overlapping acute and inflammatory stages of wound healing based on molecular, cellular, and bio-compatibility issues. In this paper the issues addressed are uncontrolled hemostasis and inflammation which can interfere with the orderly flow of wound healing. In this regard, we review the serine proteases thrombin and elastase relative to dressing functionality that improves wound healing and examine the effects of charge in cotton/cellulosic dressing design on thrombin production and elastase sequestration (uptake by the wound dressing). Thrombin is central to the initiation and propagation of coagulation, and elastase is released from neutrophils that can function detrimentally in a stalled inflammatory phase characteristic of chronic wounds. Electrokinetic fiber surface properties of the biomaterials of this study were determined to correlate material charge and polarity with function relative to thrombin production and elastase sequestration. Human neutrophil elastase sequestration was assessed with an assay representative of chronic wound concentration with cotton gauze cross-linked with three types of polycarboxylic acids and one phosphorylation finish; thrombin production, which was assessed in a plasma-based assay via a fluorogenic peptide substrate, was determined for cotton, cotton-grafted chitosan, chitosan, rayon/polyester, and two kaolin-treated materials including a commercial hemorrhage control dressing (QuickClot Combat Gauze). A correlation in thrombin production to zeta potential was found. Two polycarboxylic acid cross linked and a phosphorylated cotton dressing gave high elastase sequestration.

## 1. Introduction

The Wound Healing Cascade: Thrombin in Hemostasis and Neutrophil Proteases in Inflammation: The healing cascade which begins immediately following injury has been characterized as including four major overlapping but complex phases including hemostasis, inflammation, proliferation, and remodeling. Completion of the orderly flow of these phases of wound healing normally occurs in three weeks [1]. This paper addresses the design of biomaterials which aim at restoring normal wound healing during the hemostatic and inflammatory phases.

### 1.1. Hemostasis

Thrombin generation is closely regulated to locally achieve rapid hemostasis after injury without causing uncontrolled systemic thrombosis. Thrombin occupies a central role in clotting since it is the last in a series of serine proteases in the clotting cascade that converts fibrinogen to fibrin in the coagulation cascade. Coagulation as viewed from the conventional Waterfall/Cascade model [2] is composed of two separate pathways, and these are a function of the biochemical cascade of zymogen serine proteases being activated in succession as clotting factors wherein an extrinsic pathway (from within the tissue as a consequence of trauma *i.e.*, tissue factor) and an intrinsic pathway (unphysiologic surface activated, *i.e.*, phospholipids) culminate in the conversion by thrombin of fibrinogen to fibrin. On the other hand regulation of thrombin generation as viewed by initiation and propagation of coagulation [3] involves an initial hemostatic response triggered by tissue factor (thromboplastin). Within the vascular endothelium activated Factor VII binds to tissue factor (TF) to activate Factor X to fXa, which generates trace amounts of thrombin (0.1–1 nm). Coagulation then only precedes at high enough TF to overcome tissue factor pathway inhibitor [4]. Factor VIIa patrols the circulation in search of sites of vascular damage and trace quantities of fXa and thrombin “alert” for potential vascular damage. Propagation of coagulation centers on circulating platelets which must be of sufficiently high enough concentration in the vessel wall (7,100 platelets/μL/day) to contribute to localized thrombus formation. A platelet plug in the vascular endothelium is formed through adherence to collagen-von Willebrand factor via their glycoprotein (GP) Ib receptors. Thrombin-activated platelets play a pivotal role in processes in sustaining the procoagulant responses (the intrinsic pathway). The components needed for activated platelets to locally generate thrombin concentration for fibrinogen's efficient fibrin formation include; (1) 12,000 copies of GPIIb/IIIa receptors that concentrate fibrinogen [5]; (2) factor XIII activation by thrombin to fXIIIa which rapidly cross-links fibrin monomers [6]. Thus, fibrinogen and Factor XIII are final thrombin substrates that play pivotal roles in stabilizing the primary hemostatic plug. Formation of the hemostatic plug is modulated by the inflammatory phase of wound healing through the binding of fibrin to neutrophils which release proteases and along with the fibrinolytic system leads to vascular restoration through removal of the thrombi.

### 1.2. The Relation of Material Surface Charge to Hemostatic Activity

There has been considerable research on the effects of charged surfaces on coagulation. It is interesting that the prothrombin time (PT) and activated partial thrombin time (aPTT) assay used to detect blood abnormalities were improved upon by adding a charged contact activator like kaolin, celite or ellagic acid [7]. In the presence of a contact activator the serine proteases of the intrinsic pathway are activated in descending order of Factor XIIa-Xia-IXa-Xa. Furthermore the ability of negatively charged surfaces like glass or the mineral kaolin to initiate contact activation of FXII has been known for over 50 years [8,9]. The term ‘glass effect’ describes the observation that blood clots faster with polar surfaces than with nonpolar surfaces. Both negatively and positively charged surfaces have been known to activate different serine protease coagulation factors. For example negatively charged polyphosphate species are capable of enhancing fibrin clot structure [10], and positively charged materials have the ability to activate FVII [11]. It has also been shown that amine polymers like poly-lysine are capable of enhancing the activation of FVII, FX and FII [12].

The surface charge of materials that interact with blood is also relevant in extracorporeal, implantable, and nonimplantable biomaterial surfaces. Historically, the strong anionic nature of heparanized surfaces which also occurs in the vascular tissue [13] has been thought to promote anticoagulant activity on extracorporeal materials [14,15,16,17]. Bone implants containing hydroxyapatite, which has been shown to promote healing through platelet activation, have been examined considerably for their role in hemostatic activity [18,19,20]. In addition it has been recently noted that charge differences in metal oxides can play a decisive role in determining procoagulant and anticoagulant activity and that basic metal oxides with an isoelectric point above the pH of blood are anticoagulant while acidic metal oxides with an isoelectric point below the pH of blood are procoagulant [21]. Moreover, mesoporous bioactive glass has been found to be desirable for several categories of wound healing due to its biocompatible composition of adjustable Silicon/calcium ratios, where the material can perform as a rapid-acting hemostatic agent (high Si/Ca ratio; bioactive glass 60), a bone-generating substrate (lon Si/Ca; bioactive glass 60), and as an anticoagulant (no silica present; hydroxyapatite) [21,22].

### 1.3. Hemorrhage Control Dressings

The endpoint of hemostasis may be defined by the conversion by thromboin of fibrinogen to fibrin and/or platelet plug formation in the endothelium as described above. However hemorrhage can occur, when blood volume breaches the endothelium without sufficient thrombosis to sustain successful blood vessel sealing and clot formation in a wound. Trauma induced hemorrhage is the leading cause of preventable death on the battlefield. In recent years the U.S. Army Institute for Surgical Research (USAISR) and the Uniformed Services University of the Health Sciences has outlined ideal properties and evaluated battlefield dressings which have shown some efficacy in hemorrhage control [23,24]. The dressings evaluated by the USAISR were the Army Field Dressing (a cotton product of long-standing use), Quikclot, HemCon, and Fibrin Sealant. The Army Field Dressing, which is the standard field dressing used by the military consists of two layers of gauze that wrap densely packed cotton. It absorbs a large volume of blood, and the cotton strands stimulate platelet aggregation. Fibrin Sealant, which consists of fibrinogen and thrombin, is quite effective but the prohibitive price ($500–$1,000 per dressing) prevents widespread deployment of this type of dressing. Among some of the most successful dressings for halting lethal hemorrhagic events are metal oxides as discussed above. For example, Quikclot is a granular mineral zeolite that rapidly absorbs water in an exothermic reaction [23]. Some improvements on zeolite-impregnated dressings in the form of the kaolin-impregnated gauze (Quikclot Combat Gauze) were made, and bentonite also rapidly halts clotting [25,26]. HemCon, which is principally chitosan has strong tissue adhesive properties that seal the wound and stops bleeding through promotion of platelet aggregation.

### 1.4. Inflammation and Chronic Wounds

During the coagulation phase following injury platelets initiate healing through the release of growth factors and cytokines which recruit inflammatory cells to the wound, and activate immune cells, extracellular matrix deposition, collagen synthesis and keratinocyte and fibroblast proliferation. It is noteworthy that skin substitutes and certain carbohydrate-based wound dressings including alginate [27], DEAE sephadex [28,29], honey [30,31], and aloe vera [32] have been reported to enhance this phase of wound healing by stimulating growth factors and cytokines secreted by macrophages/monocytes, platelets, and fibroblasts, important to wound healing. Neutrophils arrive early on marking the onset of the inflammatory phase and clearing the wound of bacteria and cellular debris, which in the acute wound only lasts a few days [1]. However in the chronic wound the period of growing neutrophil population is extended indefinitely. Neutrophils mediate a variety of chemotactic, proteolytic, and oxidative events that have destructive activities in the chronic wound [33]. Hence therapeutic interventions have been proposed based on the proteolytic and oxidative mechanisms of neutrophil activity in the wound. Neutrophils contain both matrix metalloproteases and cationic serine proteases, which are two classes of proteases associated with a variety of inflammatory diseases. The presence of elevated levels of these proteases in non-healing wounds has been associated with the degradation of important growth factors and fibronectin necessary for wound healing in addition to oxidation of protease inhibitors which leads to unchecked protease activity. We and others have approached this pathology by developing both elastase inhibitor-released dressings, and elastase sequestrant dressings [34,35,36,37,38,39,40].

### 1.5. Negatively Charged Materials for Chronic Wounds

The design of anionic materials that bind cationic elastase from wound fluid is based on presentation by the cellulose fiber of a negative counter ion at wound pH which binds the cationic serine protease and thereby removes the protease through an ion exchange mechanism. The large amount of cationic serine protease released into the chronic wound is a result of the high titer of elastase present in neutrophils, with as much as one picogram of elastase stored in a single neutrophil granule. We and others have explored the use of negative counter ions to bind and sequester elastase and similar cationic serine proteases released into the chronic wound. For example sulfonation, carboxymethylation, and phosphorylation finishes on cotton dressings are effective in removing elastase [35]. A sulfonated polymer hydrogel also removes elastase from the wound bed [41]. Interestingly carboxy methyl benzylamide sulfonate dextran previously has been shown to inhibit elastase, and may utilize ionic binding to facilitate enzyme uptake [42].

### 1.6. Biocompatibility of Cellulosics and other Materials and the Role of Charge

Issues in controlling the interactions between blood and wound proteins at the biological interface of biomaterials including non-implantable, implantable or extracorporeal materials are complex. Among these are understanding the role of interfacial free energy, wettability (hydrophilic *versus* hydrophobic balance) [43,44], surface charge [45,46,47], surface patterns [48,49,50,51], and molecular size or conformation [46] to name some important in design. As a biomaterial underivatized cellulose is very hydrophilic [50,51] with a relatively high surface energy regarding moisture uptake but a relatively low interfacial free energy with regard to its ability to imbibe water and reduce protein absorption. Cellulose also varies in surface charge, fiber surface length and pattern depending on its source [52,53,54]. The materials of this study are characteristic of this variation. In addition, cellulose-based materials continue to be widely used in extracorporeal, implantable, and non-implantable medical devices. For example cellulose materials have long been used in wound dressings [55], are used in 80% of the dialyzers with very good permeability for low molecular weight substances [56], and are of increasing interest in tissue engineering [57]. Modified cellulose materials have been widely used in a variety of wound healing pathologies. These include materials to halt blood flow [23] and to treat non-healing wounds for absorbing excessive exudate, debridement, and sequestering proteases [34,35,36,37,38,39,40,51,58,59].

### 1.7. Modified Cotton Dressings for Hemostasis and Chronic Wounds

Bleached and scoured cotton as is produced in medical woven cotton gauze is ninety-nine percent cellulose [60]. Since ancient Greece cotton has long been used in wound dressings [61], and is still a standard of comparison when developing new hemorrhage control dressings for hemostatic activity [62]. We have recently reported the use of positively and negatively charged cotton wound dressing on two stages of wound healing—hemostasis and inflammation [63]. This paper further reports the relative effects of the fiber surface charge of cotton, chitosan grafted onto cotton, and a kaolin-containing dressing on thrombin production. In addition negatively charged polycarboxylic acid cross-linked derivatives of cotton dressings were evaluated for elastase sequestration. The design and preparation of polycarboxylic acid cross-linked cotton as an elastase sequestrant is based on presentation of a negatively charged substrate to bind the cationic serine protease elastase in the chronic wound. Human neutrophil elastase is rich in arginine residues on the surface of the protein and available for interaction with acidic polysaccharides as are found in the azoruphil granule of neutrophils where it is released into the chronic wound in high concentration. Thus a series of polycarboxylic acid crosslinked cotton analogs were prepared and evaluated for elastase sequestrant activity [63]. In a similar manner the design and preparation of phosphorylated cotton gauze was based on the ability of negatively charged phosphorylated gauze to bind positively arginine side chain residues of elastase.

### 1.8. Electrokinetic Assessment of Material Surface Charge

As discussed above, the surface charge of materials may be characterized by their zeta potential or the zeta potential plotted according to pH titration, and an understanding of the materials charge at the pH of an acute or chronic wound is relevant to a picture of the role of charge in hemostasis. The ζ_plateau_ derived from the zeta potential pH titration also reveals the relative hydrophobicity or hydrophilicity of the fiber [64,65]. The surfaces of materials may be characterized by their zeta potential. Due to the historic importance of interface properties between hydroxyapatite with biological fluids considerable work on correlating electrokinetic properties with surface charge and substrate function have been characterized. For fibrous samples like the ones employed in this study the maximal zeta potential of the fiber surface generally occurs in the alkaline range indicating the fiber's hydrophilic or hydrophobic behavior [64,65]. The ζ_plateau_ from an electrochemical pH titration reveals the relative hydrophobicity or hydrophilicity of the fibeṛ. A pronounced plateau, zeta plateau (ζ _plateau_), is observed for the materials studied here. Zeta potential measurements were made of the hemostatic and elastase sequestration materials of this study to assess the relative fiber surface properties of charge, hydrophilicity and hydrophobicity.

Zeta potential is described here according to the double layer model of Gouy-Chapman-Stern-Grahme model for negative surface charge [66,67,68]. This model can be described as two ionic planes (anionic and cationic) which present as the electrochemical double layer of the solid surface. The cationic layer is designated as the inner Helmholtz plane (IHP) and the anionic layer as the outer Helmholtz plane (OHP). At an increasing distance from the solid surface is a diffuse layer of ions subject to electrostatic attractive forces from the surface of the material as well as thermal motion. Between the fixed and diffuse ions is the shear plane which corresponds to the zeta potential. Wetting (swelling) of fibers expands the inner surface of the fiber, and the electrochemical double layer migrates in this swelling layer. Consequently ions in the shear plane of the fiber shift towards the bulk electrolyte solution causing a potential drop and decrease in zeta potential. Competitive adsorption of water at the electrochemical double layer of the fiber causes the amount of ions on the surface to decrease during fiber swelling and a simultaneous decrease in zeta potential. The cellulosic wound dressings studied here conform well to this model.

## 2. Materials and Methods

Preparation of Polycarboxylic Acid Cross linked, Phosphorylated and Chitosan-Grafted Cotton Dressings.

USP Type VII cotton gauze sponges (12 ply—4 in. × 4 in.) were treated in solution pad baths consisting of 0.62 M 1,2,3,4-butanetetracarboxylic acid and 4% sodium hypophosphite, and the gauzes were padded by two repetitions of dipping in the treatment solutions followed by removal of excess solution on a laboratory mangle with about 90% wet add-on. In a similar manner 0.62 M 2-ketoglutaric acid and 1,2,3,4,5,6,-cyclohexanehexacarboxylic acid were processed onto cotton gauzes sponges. The padded fabrics of the three different polycarboxylic acid cross linking agents were dried and cured at 155 °C in ovens with mechanically circulated air. The treated gauzes were then washed under deionized water for one hour following the treatment. Preparation of chitosan-grafted fabrics was completed by pad-dry-cure using Chitosan purchased from Sigma Chemical plus 11.9% citric acid plus 4% sodium hypophosphite, on the USP Type VII cotton gauze sponges; dried at 85C for 5 minutes and cured at 160 °C for 3 min. The polycarboxylic acid crosslinked cotton gauze was confirmed by the method of Yang [69] as previously outlined [70]. FT-IR spectral analysis of the crosslinked analogs was used to show that the spectral band of the ester carbonyl can be separated from the bands of the carboxylic acid and carboxylate anion found in the cotton fiber. FTIR spectra were obtained on a total Scimitar Digilab Fourier Transform Infrared spectrometer. Spectra were obtained directly from cotton samples on a Durascope. Samples were scanned within the range of 500–5,000 wavenumbers and analyzed on Digilab Merlin software. In summary base treatment of the crosslinked fabric gives an increase in the intensity of the band at 1,585 cm^−1^ and a decrease in the 1,725 cm^−1^ band intensity occurred in the spectrum of the cotton gauze fibers. When the gauze is treated with 0.1 M HCl for two minutes at room temperature an increase in the band at 1,725 cm^−1^ occurs and the band at 1,585 cm^−1^ disappears (as shown in Figure 3D and Figure 4D). Thus the band at 1,585 observed in the spectra of the conjugates is due to the carbonyl of the carboxylate anion of the crosslinked cotton. Chitosan-grafted print cloth was completed in a similar manner to that of the polycarboxylic acid crosslinked cotton (Figure 5a). These fabrics were padded with (1%) chitosan plus 11.9% citric acid, plus 4% sodium hypophosphite; dried at 85 °C for 5 min. and cured at 160 °C for 3 min. Chitosan grafted onto cotton was characterized as a per cent add-on by weight (5%). Phosphorylated cotton was prepared as previously outlined [39]. In summary the cotton gauze were padded with a sodium hexametaphopshate:urea (10 wt.%:30 wt.%) solution, and IGEPAL-CA630 was used as a wetting agent and added as a 0.1% padding solution. The cotton gauze was dried at 90 °C for 3 min and cured at 160–175 °C for 3–7 min followed by rinsing with de-ionized water and air dried. Phosphorylated cotton was characterized with infrared analysis and phosphorous elemental analysis. Phosphorous levels based on elemental analysis were found to be 3%. These phosphorous levels are consistent with previous reports.

### 2.1. Thrombin Assay

**Figure 1:**
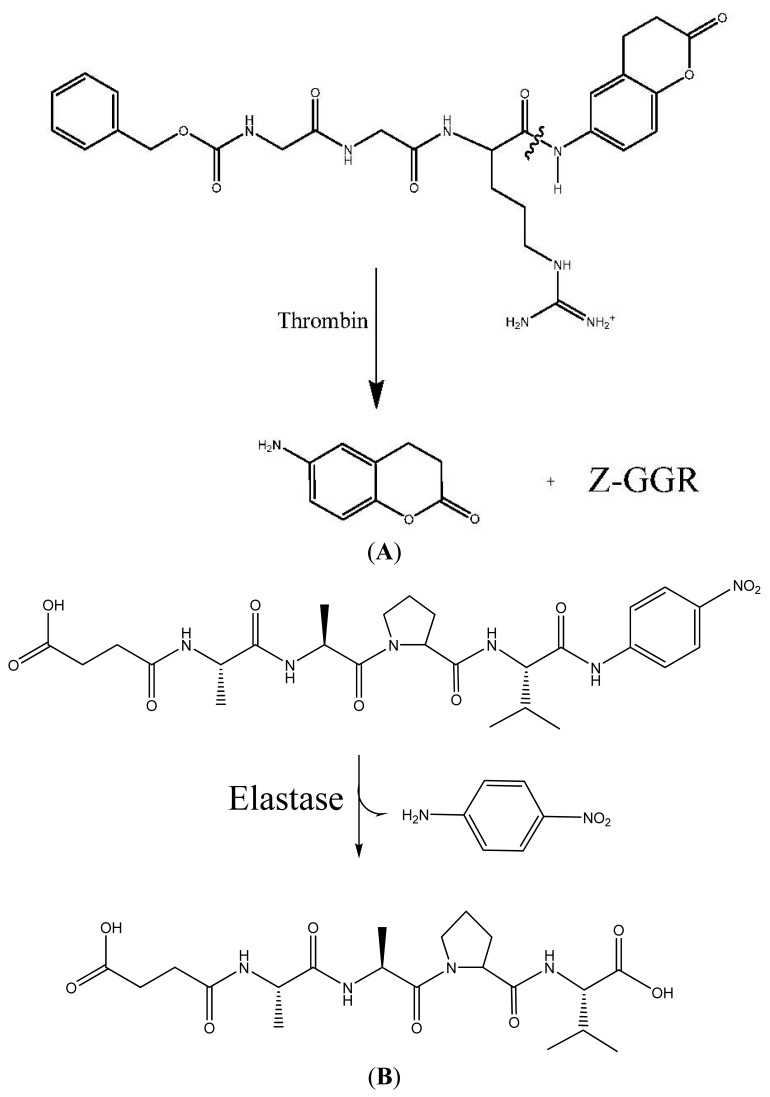
(**A**) Thrombin substrate hydrolysis: Benzoyl-glycyl-glycyl-arginyl-7-amido-4-methylcoumarin (Z-GGR-AMC) hydrolysis by thrombin through scission of the COOH-terminal amide coumarin bond to release the fluorescent 7-amino-4-methyl-coumarin; (**B**) Hydrolysis of elastase substrate Suc-Ala-Ala-Pro-Val-pNA.

An assay for monitoring blood coagulation via thrombin release and hydrolysis of a fluorogenic substrate was developed. The assay adopted from TECHNOTHROMBIN TGA is based on monitoring the fluorescence generated by the cleavage of a peptide fluorogenic substrate (Carbobenzoxy-Gly-Gly-Arg-AMC) by thrombin, as shown in Figure 1 during the clotting process. From the changes in fluorescence over time, the concentration of thrombin (nM) in the sample can be calculated using the respective thrombin calibration curve. Monitoring the increase in thrombin concentration with time allows calculating thrombin associated with each hemostatic sample and correlates the hemostatic material to thrombin values over time for the whole coagulation process. This assay approach results in the visualization of the different phases of clot formation. The assay procedure was followed as outlined in ‘TECHNOTHROMBIN TGA; for research only’ obtainable at www.technolcolone.com. The assay was adapted to a microtiter plate reader containing a fluorescence detector using a Synergy HT which was set at 390-nm excitation and 460 emission filter (BioTek: Winooski, VT, USA). Samples of kaolin, cotton, QuickClot Combat Gauze (Z Medica: Wallington, CT, USA) and chitosan-grafted cotton were pre-weighed and added to the plates prior to adding the reagents. Hepes-NaCl buffer (20 microliters) was added to the samples. A 10 microliter solution of phospholipid micelles in Tris Hepes-NaCl buffer (supplied by Technothrombin TGA as Reagent RD) was added to all wells containing samples, controls, and blanks. A 30 microliter solution of bovine plasma in 15 mM calcium chloride (Hemostat Labs; plasma was previously collected in sodium citrate at a 80:20 ratio) was added to the microtiter wells. The blanks contained reagent buffer (20 microliters), reagent RD (10 microliters) and citrated blood (30 microliters). A 50 microliter solution of the substrate Cbz-Gly-Gly-Arg-AMC (1 mM) with 15 mM CaCl_2_ was added to all the wells.

The assay was completed as prescribed and evaluation software was used to calculate thrombin generation in the samples over time and the results were given in nM thrombin concentration generated in the samples for each point of time during the whole coagulation process. Upon initiation of coagulation in the samples by addition of CaCl_2_ and the phospholipids/tissue factor mixture, generation of thrombin is initiated after a lag period; thereafter thrombin generation per minute increases, reaching a maximum of thrombin generation and decreases thereafter.

### 2.2. Streaming Zeta Potential

Streaming zeta potential experiments were carried out with the Electro Kinetic Analyzer (Anton Paar: Richmond, VA, USA) using the Cylindrical Cell developed for the measurement of fibrous samples. For each measurement a fiber plug was placed between the Ag/Gal disc electrodes of the Cylindrical Cell. The pH dependence of the zeta potential was investigated with the background electrolyte of 1 mM KCl solution. The evaluation of zeta potential is based on the Smoluchowski equation [71]:

$$\zeta =\frac{\text{dU}}{\text{dp}}\frac{\eta \kappa }{{ɛ}_{\text{r}}{ɛ}_{\text{o}}}$$

where U is the streaming potential, p the pressure, ε_ρ_ and ε_o_ the dielectric constant and the vacuum permittivity, η the viscosity and κ is the conductivity of the measuring fluid. Surface conductivity of the fibrous samples was not taken into account.

### 2.3. Elastase Assay

Treated and untreated gauze samples were submerged in 1 mL of buffer (pH 7.6 buffer composed of 0.1 M sodium phosphate, 0.5 M NaCl) containing 84 munit/mL of human neutrophil elastase. Samples were allowed to incubate for one hour at room temperature, after which they were removed and placed in a 5 mL syringe and pressed to drain unbound buffer and enzyme. The unbound elastase fractions were combined and assayed for elastase activity. Enzyme assays of the solutions containing unbound human neutrophil elastase were conducted in pH 7.6 buffer described above and subjected to spectrophotomeric measurement of the release of p-nitroaniline at 410 nm from the enzymatic hydrolysis of N-Methoxysuccinyl-Ala-Ala-Pro-Val-p-nitroanilide (Sigma) [39]. The spectrophotometric kinetic assays were performed in a Bio-Rad Microplate Reader (Hercules, CA, USA) with a 96-well format.

## 3. Results and Discussion

### 3.1. Thrombin Production to Assess Hemostatic Materials

To adequately assess the ability of a material to perform as a hemorrhage control dressing both clotting assays such as thromboelastographs and *in vitro* thrombin assessment are ideally employed. However, since increased rates of substrate hydrolysis by thrombin correlate with the coagulability of the sample, assessment of the effects of the materials of this study on thrombin production during coagulation was performed [72]. As discussed above, the central role of thrombin in coagulation justifies monitoring thrombin activity at the real-time coagulability of the sample. Thrombin activity at real-time coagulability of the samples is also at the pH of the acute wound [73]. In Figure 1 the reaction cascade of thrombin acting as a serine protease and the fluorogenic peptide substrate of thrombin are shown. Release of the fluorescent 7-amino-4-methylcoumarin, upon hydrolysis by thrombin at the COOH-terminal amide of the peptide, allows monitoring the rate of thrombin production during coagulation of whole blood. Increased rates of substrate hydrolysis by thrombin correlate with the coagulability of the sample. It is noteworthy that the benefit of a fluorogenic assay is that the fluorescence signal is only minimally affected by adsorption due to the blood or the appearance due to a clot and turbidity and the sample material. In addition fibrinogen does not need to be removed, and platelet aggregation does not affect the fluorescence signal [74].

**Figure 2:**
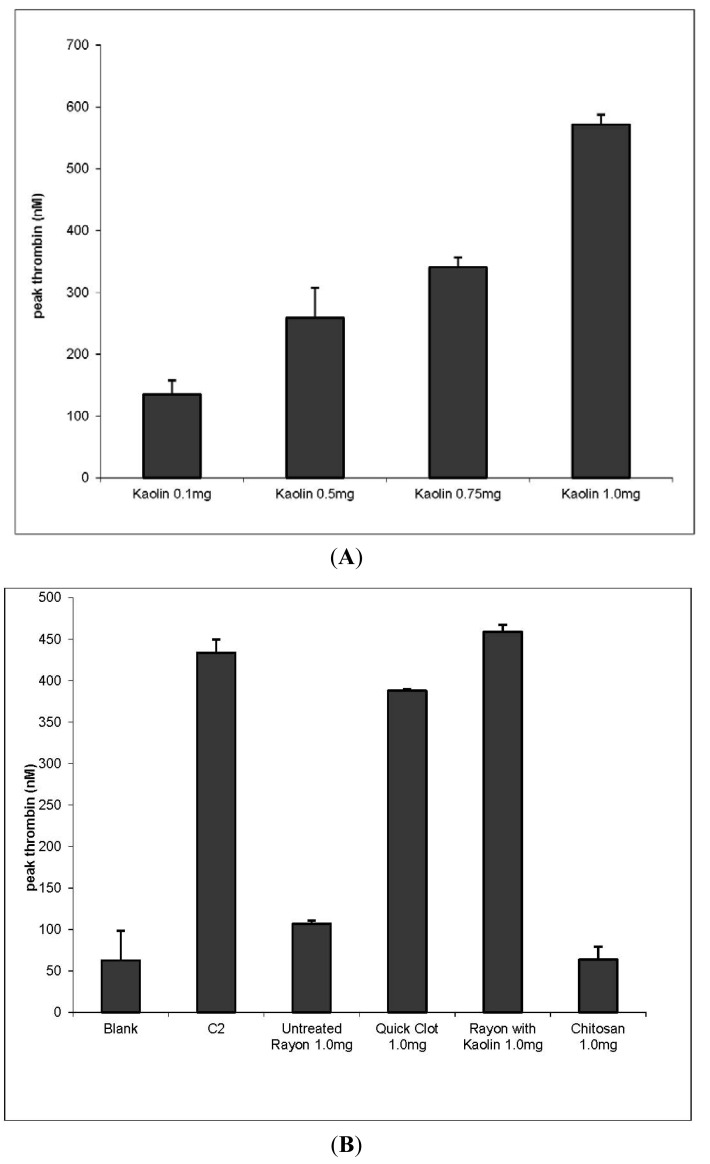

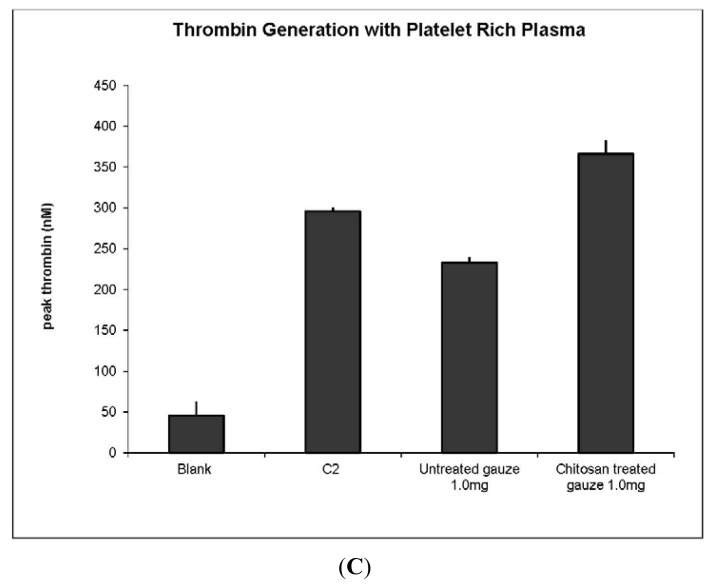
(**A**) Thrombin dose response using the clay mineral kaolin in platelet rich plasma; (**B**) Thrombin concentrations elicited by QuickClot and rayon/polyester-dusted kaolin at a similar dose of kaolin in platelet rich plasma. This demonstrates the relative thrombin production of the dressing with impregnated kaolin *versus* kaolin dusted on the surface; (**C**) Thrombin concentration elicted by chitosan-treated cotton gauze in platelet rich plasma relative to untreated cotton gauze in platelet rich plasma.

To assess the comparative effects of dressings of this study on thrombin production an analysis was made of dressing materials included in QuickClot Combat Gauze (QC), which is a rayon/polyester gauze with 10% kaolin impregnated, and a chitosan grafted cotton gauze and sponge. The clotting profile observed with kaolin as seen in Figure 2A demonstrates a linear dose response within a similar concentration range of up to 500 nM thrombin production at 1mg of kaolin. Since (QC) contains kaolin, the carrier materials (rayon/polyester) were compared for their thrombin production independent of the dressing as shown in Figure 2B. The results of thrombin production indicate that (QC) yields higher thrombin production than the rayon/polyester dressing. When kaolin is dusted on the surface of the rayon/polyester gauze at the same weight ratio as found in Quickclot the thrombin response is increased. This finding when compared with kaolin alone demonstrates the relative role of kaolin to increase thrombin production. An increase in thrombin production in the kaolin-dusted dressing may be a result of the more rapid contact of kaolin with blood. This finding is consistent with recent reports on the mediation of hemostasis by metal oxide surface charge [21].

Because of the potential to alleviate major hemorrhaging and improve on the fabric integrity of chitosan-based textiles we have incorporated chitosan into a variety of woven and non-woven fabrics and shown the enhanced ability of chitosan-treated cotton fabrics to promote blood clotting [60,75,76,77,78,79]. Chitosans have been reported to promote rapid hemostasis and prevent massive venous hemorrhage [75]. In Figure 2C is shown the results of chitosan clotting using platelet rich plasma to monitor thrombin generation by chitosan-grafted gauze. The results demonstrated increased production of thrombin by the chitosan-grafted cotton gauze when compared with untreated cotton gauze. Since chitosan initiation of clotting is marked by platelet aggregation this is not unexpected. Moreover increased thrombin production in platelet poor plasma was not observed (unpublished results). However, the use of chitosan-grafted gauze may pose some advantages due to cottons enhanced absorbance properties and the potential to further derivatize the cotton cellulose to improve on hemorrhage control. Chitosan has been extensively studied for its effects on wound healing, and activation of hemostasis [78]. However, the hemostatic responses to the chitosans have been found to be highly dependent on their chemical nature and tertiary/quaternary structure [79]. Chitin microfibers have been shown to activate platelets and increase the formation of the fibrin matrix [80], and the activation of the intrinsic coagulation cascade has shown a marked increase in thrombin production when chitin interacts with red blood cells [81].

### 3.2. Elastase Sequestration by Negatively Charged Cotton Dressings

**Figure 3:**
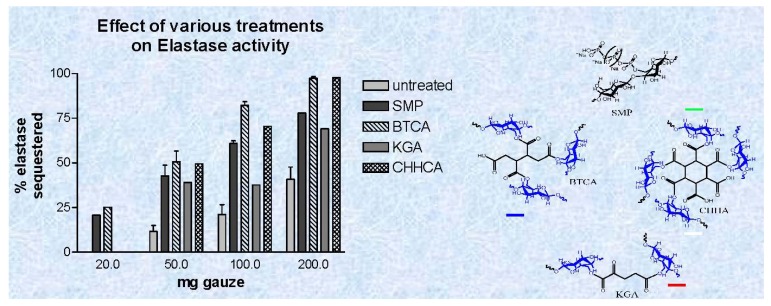
A dose response plot of activity reduction of elastase *versus* weight of cotton wound dressing for the phosphorylated and polycarboxylic acid cross linked cellulose dressings. The phosphorylated cellulose dressings were treated with urea and sodium hexametaphosphate-treated (SMP) and dominium phosphate (DAP), and the polycarboxylic acid cross linked dressings were reacted with1,2,3,4-butanetetracarboxylic acid (BTCA), 2-ketoglutaric acid (KGA), 1,2,3,4,5,6,-cyclohexanehexacarboxylic acid and untreated gauze. Data are the mean ± SD of triplicate determinations. The method and conditions for the assay are given in the Materials and Methods section.

We have previously shown that elastase sequestration can be affected through several functional motifs incorporated into cotton including peptide-cellulose conjugates, dialdehyde, carboxymethylated, sulfonated, and phosphorylated cotton [55]. Thus, from this work it has been shown that charged dressings can be developed that remain effective in removing human neutrophil elastase from wound fluid as it is produced in chronic wounds over a 24 h period. We report in this paper for the first time the use of polycarboxylic acid crosslinked cotton to remove elastase from wound fluid. Figure 3 shows both the structures and a dose response profile of three types of polycarboxylic crosslinked cottons compared with untreated and phosphorylated cotton, which was previously reported. The polycarboxylic acid crosslinked cotton analogs were characterized with IR as previously described. Previously Yang *et al.* [69] showed that citrate ester crosslinkages in cotton cellulose can be distinguished from the corresponding citrate acid and carboxylate anions through IR analysis of acid and base treated fabric. FT-IR spectral analysis of the glucose and fructose conjugates was used to show that the spectral band of the ester carbonyl can be separated from the bands of the carboxylic acid and carboxylate anion found in the cotton fiber. Butane-tetra-carboxylic acid and cyclohexane-hexacarboxylic acid cross linked cotton removed 50–90 percent of elastase from solutions of mimicked chronic wound fluid, and phosphorylated cotton removed 40–70 percent of elastase; whereas, ketoglutaric acid removed 35–60 % from the solution.

### 3.4. Zeta Potential Measurements on Hemostatic and Chronic Wound Dressings

Zeta potential measurements were determined for the hemostatic and elastase sequestrant materials of this study. The materials assessed included cotton gauze, chitosan-grafted cotton gauze, kaolin-dusted cotton gauze and the commercial dressings including QuickClot Combat Gauze.

**Figure 4:**
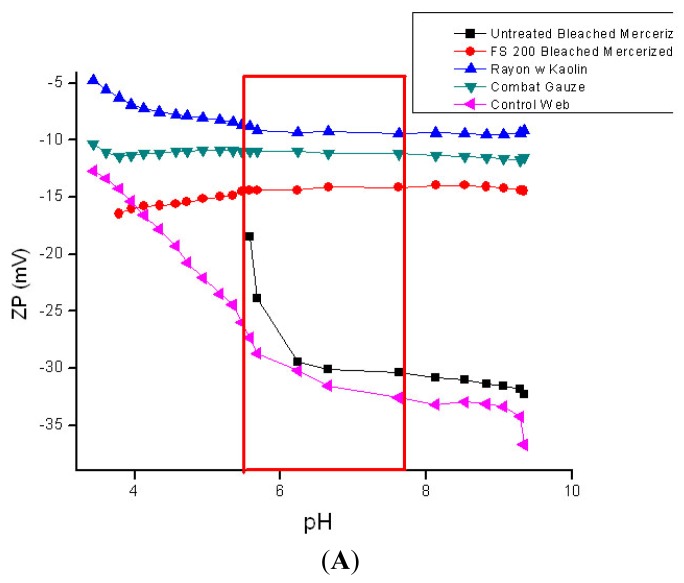

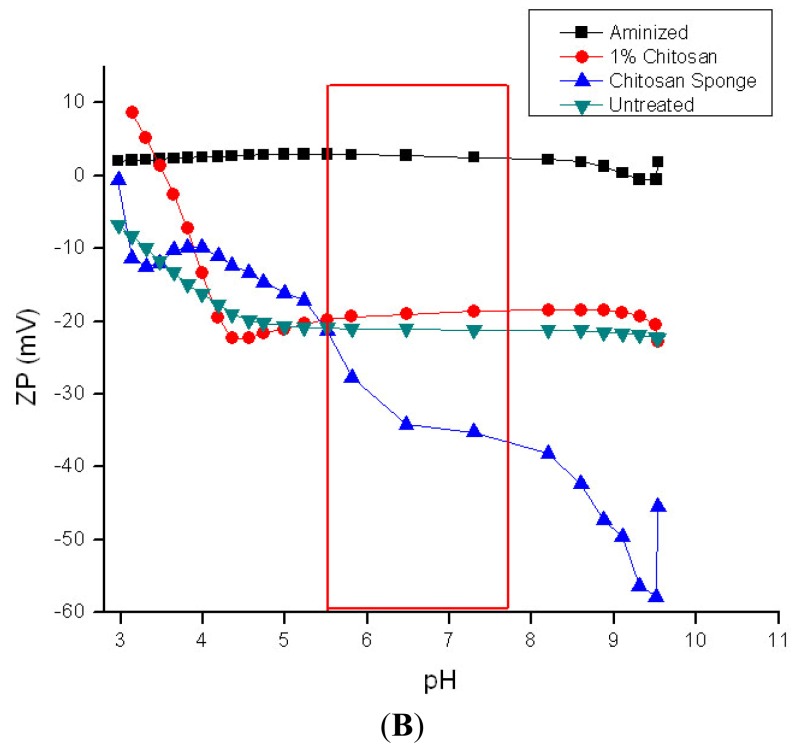
(**A** and **B**): Streaming Zeta potential titrations of hemostatic materials. Streaming zeta potential titrations performed as outlined in the Materials and Methods section. The outlined box encloses the pH range of the acute wound; (**A**): ■ needlepunched nonwoven cotton; ▲ rayon/polyester gauze dusted with kaolin; 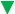 QuickClot Combat Gauze (4A); 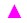 Rayon/polyester, 50/50 gauze; (**B**) ■ aminized cotton gauze; 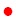 chitosan-citric acid grafted cotton gauze; 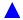 chitosan-lactic acid composite sponge; 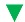 untreated cotton gauze (4B).

The pH of the acute wound at hemostasis is approximately 6.2, and it becomes more acidic during the inflammatory stage of wound healing, steadily increasing during granulation and returning to normal during the final stages of re-epithelialization [73]. Whereas, the pH of chronic wounds which are arrested in the inflammatory stage of wound healing average around 7.5 with considerable variation. An advantage of zeta potential pH titration as shown in Figure 4 A and B is that it allows assessment within the wide range of pH found in wounds. Previously it has been suggested that the sign and magnitude of the zeta potential for hemostatic materials be measured in the presence of CaCl_2_ electrolyte that mimics the calcium ion concentrations found in blood serum [21]. This type of study will be the subject of future work with these materials. Here we report the zeta potential charge from pH titrations for comparison of the materials tested for thrombin production and elastase sequestration.

**Figure 5:**
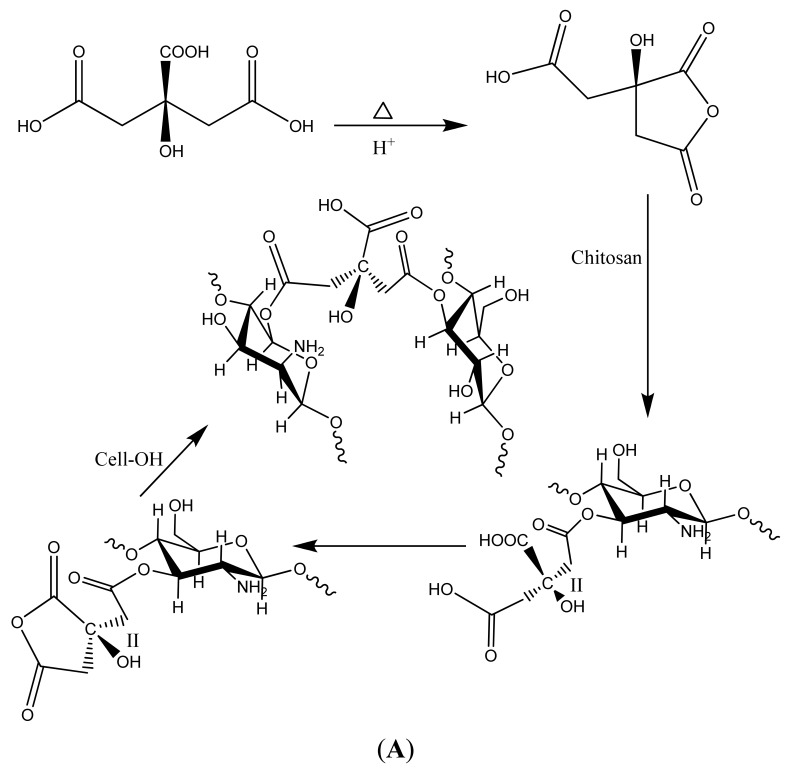

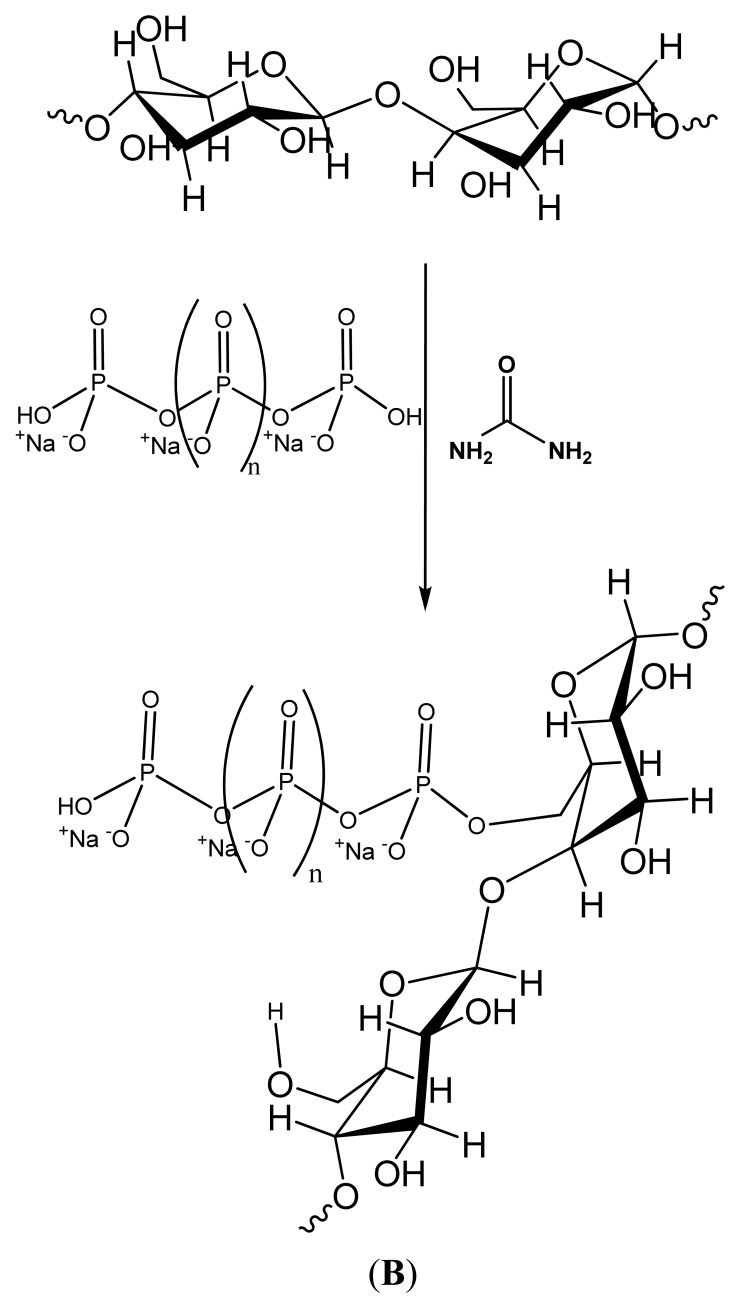
(**A**) Synthetic pathway of chitosan-grafted gauze using citric acid, acid catalyzed crosslinking between chitosan and cellulose; (**B**) Phosphorylation of cotton cellulose [82].

As shown in Figure 4A the kaolin treated materials gave a ζ_plateau_ that was more indicative of very hydrophilic character (ζ_plateau_ = −7 mV). Whereas the untreated cotton gauze and rayon/polyester dressing had considerably more negative ζ_plateau_ (−32 mV) values, and thus are deemed to be more hydrophobic relative to the kaolin treated rayon/polyester and Quickclot Combat gauze. In Figure 4B the chitosan sponge which contains lactic acid showed a ζ_plateau_ at −35 mV at pH 7.5. Aminized cotton was selected to compare with chitosan since there are structural and molecular similarities in the glucosamine charge. Aminized cotton has a slightly positive ζ_plateau_ (+3 mV), which is constant within a wide acidic and alkaline pH range, including those in the pH range of the acute wound [73]. Aminized cotton was also isoelectric or neutral throughout the entire titration. These properties should combine to make it highly adhesive. However, aminized gauze in this study proved difficult to measure for thrombin production, and did not give consistent results. Chitosan was grafted onto cotton cellulose, and the reaction used to prepare chitosan-grafted cotton is shown in Figure 5A. Chitosan-grafted cotton demonstrated a negative ζ_plateau_ around −20 mV at pH 6; however, chitsoan-grafted cotton gauze was only slightly more hydrophilic than untreated cotton. This electrokinetic profile for chitosan-grafted cotton is consistent with the presence of citrate carboxylate groups and the glucosamine residues of chitosan, which confer both a negative and positive charge respectively (see structure of crosslinked of chitosan and cellulose in Figure 5A).

**Figure 6:**
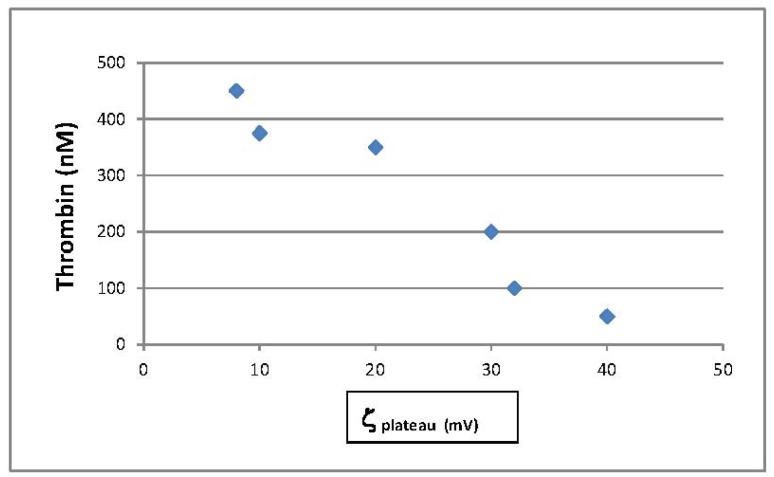
Thrombin production (nM) *versus* ζ_plateau_ (mV) as taken from the zeta potential titrations found in Figure 4 and Figure 7 for the materials of this study.

Figure 6 contrasts thrombin production by the hemostatic materials with the corresponding ζ_plateau_ found (plot of ζ_plateau_ *versus* thrombin concentration). It is interesting that there is a correlation in this study between thrombin production and the ζ_plateau_. As the material's become more hydrophilic *i.e.*, a ζ_plateau_ that approaches neutrality, the observed thrombin production increases. However other factors including surface area and heterogeneity may also play a role in the thrombin production [50,51,52,53,54]. In addition, as discussed above negatively charged substrates activate the intrinsic pathway of the coagulation cascade [7,8,9,10,13,14,15,16,17]. Thus additional property considerations are required to understand whether the correlation between ζ _plateau_ and thrombin production found here is meaningful in terms of coagulability of the materials.

**Figure 7:**
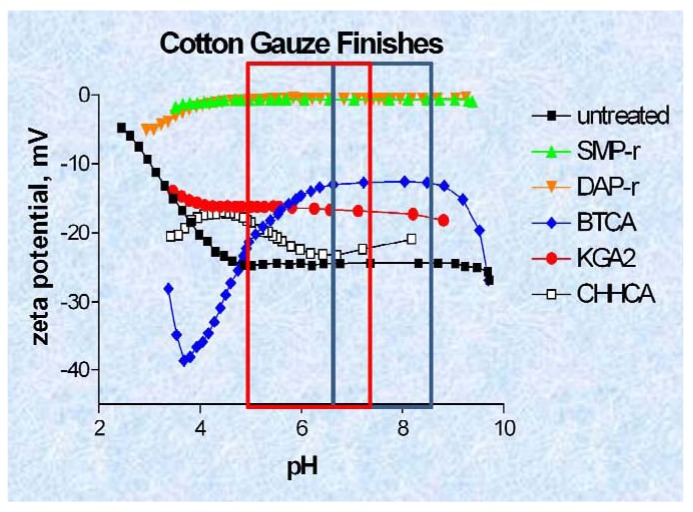
Streaming Zeta potential titrations of hemostatic materials and chronic wound dressings. Streaming zeta potential titrations performed as outlined in the Materials and Methods section. The outlined box encloses the pH range of the acute and chronic wounds; A: ■—untreated cotton gauze; 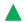 and 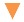 Phosphoryoated cotton gauze; 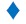 1,2,3,4-butanetetracarboxylic acid (BTCA), 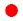 2-ketoglutaric acid (KGA; 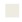 1,2,3,4,5,6,-cyclohexanehexacarboxylic acid.

Figure 7 shows the zeta potential titrations of the elastase sequestration dressings. The order of hydrophilicity for the dressing was SMP > BTCA > KGA > CHHA = untreated. Since the BTCA and CHHA were the two most effective elastase sequestrants observed in this study there aren't any apparent correlations between ζ_plateau_ and elastase sequestration among the polycarboxylic cross-linked and phosphorylated dressings. This may in part be due to the hydrophobicity contributed by the cyclohexane rings in CHHA. However it is noteworthy that most of the materials and especially the phosphorylated cotton (chemistry to prepare the phosphorylated cotton is shown in Figure 5B) demonstrate a constant zeta potential within the pH range that occurs in the chronic wound.

## 4. Summary

We have reviewed some of the salient issues native to hemostasis and inflammation regarding biomaterial charge, and reported some of our recent findings on thrombin production and elastase sequestration as a function of the electrokinetic profile of modified cellulosic materials. By combining considerations of the hemostatic and inflammatory stages of wound healing in these two serine proteases with material design, it is hoped that future work will necessarily consider the overlap and design of wound healing materials, *i.e.*, interactions of inflammatory proteases with fibrin to name one example [83]. More work on the tunable functional features of dressings such as mixed charged materials, which promote both hemostasis and attract growth factor-containing macrophages, may lead to improved dressings [84]. In addition revisiting the mechanism of action of controlled release molecules like oleic acid which has been found to both accelerate wound closure and inhibit inflammatory proteases like elastase, presents an emblematic approach to improving wound healing materials [32,85]. Our approach of focusing on cellulosic dressings for wound healing as reported here may also enable the development of more cost effective dressings where patient management of chronic wounds is needed to remove excess proteases. In this regard a phosphorylated cotton dressing which would be relatively low cost has received recent FDA approval to be used in chronic wounds [39,82].
